# New Drugs, Appliances, and Things Medical

**Published:** 1893-02-11

**Authors:** 


### NEW DRUGS, APPLIANCES, AND THINGS MEDICAL.

[All preparations, appliances, novelties, etc., of which a notice is desired, should be sent for the Editor, to care of The Manager, 140, Strand, London, W.C.]

### SAFETY LAMPS.

Messrs. A. C. Wells, of Manchester and London, deserve gratitude from the public for placing within the reach of all the most serviceable of safety lamps. The cheapness of paraffin and benzoline cause both to be largely used by the poorer classes. The danger arising from the use of ordinary lamps, especially to children, is only too apparent by the constant occurrence of fatal accidents. By the use of Wells's lamps either for benzoline or paraffin the dangers are entirely removed. The lamps are made of heavy metal, with a wide, flat base, and are thus difficult to upset. Further, material placed within the body of the lamp entirely absorbs the liquid, so that there is practically nothing to upset. The specimen we have received is a small benzoline lamp, which we tested in every way, and found to fulfil all the merits claimed for it. This lamp has no globe, but others can be obtained with this supplied at 2s. each, and would be found preferable, if required, as a hand lamp. Messrs. Wells supply both benzoline and paraffin lamps at even lower prices than those mentioned, so that a trial is not beyond the smallest means, and we cannot too strongly urge their adoption on the score of safety. The "Cleanly" Lamp Filler, also manufactured by this firm, is a really excellent contrivance to obviate one of the greatest objections to the use of lamps. The filler is in the shape of an ordinary oil can, provided with a pump and delivery pipe, which when charged can be regulated to a single drop, and as no spilling is possible, the disagreeables of lamp-cleaning are reduced to a minimum.

